# Mutations in Two *Paraburkholderia phymatum* Type VI Secretion Systems Cause Reduced Fitness in Interbacterial Competition

**DOI:** 10.3389/fmicb.2017.02473

**Published:** 2017-12-12

**Authors:** Samanta Bolzan de Campos, Martina Lardi, Alessia Gandolfi, Leo Eberl, Gabriella Pessi

**Affiliations:** Institute of Plant and Microbial Biology, University of Zurich, Zurich, Switzerland

**Keywords:** *Rhizobium*, legume, symbiosis, nodulation, competitiveness, T6SS, biofilm

## Abstract

*Paraburkholderia phymatum* is a highly effective microsymbiont of *Mimosa* spp. and has also been shown to nodulate papilionoid legumes. *P. phymatum* was found to be highly competitive both in a natural environment as well as under controlled test conditions and is more competitive for nodulation over other α- and β-rhizobial strains in a variety of different plant hosts. In order to elucidate the factors that make this bacterium highly competitive for legume infection, we here characterized the type VI secretion system (T6SS) clusters of *P. phymatum*. T6SSs have been shown to function as a contact-dependent injection system for both bacterial and eukaryotic cells. We identified two T6SS clusters in the genome, created respective mutant strains and showed that they are defective in biofilm formation and in interbacterial competition *in vitro*. While the T6SS mutants were as efficient as the wild-type in nodulating the non-cognate host *Vigna unguiculata*, the mutants were less competitive in *in planta* competition assays, suggesting that the T6SS is one of the factors responsible for the success of *P. phymatum* in infecting legumes by directly inhibiting competitors.

## Introduction

β-proteobacterial strains of the genus *Burkholderia* are found in a variety of environments and can be pathogenic (e.g., *B. pseudomallei*, *B. thailandensis*, *B. cepacia*, *B. cenocepacia, B. glumae*, and *B. gladioli*) or beneficial (e.g., *B. phytofirmans, B. tuberum*, and *B. phymatum*) to a wide range of eukaryotic hosts (Suarez-Moreno et al., 2012; Estrada-de los Santos et al., 2013; Eberl and Vandamme, 2016; Beukes et al., 2017). The discovery of β-proteobacteria (from the *Burkholderia* and *Cupriavidus* genera) inside root nodules of a leguminous plant in 2001 (Moulin et al., 2001) gave rise to a new class of rhizobia (the so called β-rhizobia) and highlighted the variety of bacteria present in the soil and in root nodules. Several studies that have followed the first description have rapidly increased the number of novel β-rhizobia, (Vandamme et al., 2002; Chen et al., 2003; Bontemps et al., 2010; Gyaneshwar et al., 2011). These new β-rhizobia are mainly found associated with *Mimosa* species from South America and Asia (Chen et al., 2005, 2006, 2007, 2008; Elliott et al., 2007b; dos Reis et al., 2010; Mishra et al., 2012); however, some species like *B. phymatum* and *B. tuberum* are able to enter symbiosis with papilionoid legumes such as those found in the South African fynbos, common bean (*Phaseolus vulgaris*) and siratro (*Macroptilium atropurpureum*) (Elliott et al., 2007a; Garau et al., 2009; Talbi et al., 2010; Angus et al., 2013; Beukes et al., 2013; Howieson et al., 2013; Lemaire et al., 2016). In the last few years, several plant-beneficial *Burkholderia* strains have been reclassified into the new genus, called *Paraburkholderia* (Sawana et al., 2014; Beukes et al., 2017). Most of the previously identified *Burkholderia* species that induce the formation of nodules and are able to fix nitrogen as symbionts are now referred to as *Paraburkholderia*.

Similarly to α-rhizobia, β-rhizobia have diverse host range and environmental preferences (Gyaneshwar et al., 2011). An important aspect of bacterial communities in the soil is the competition between the different species. Previous studies for nodule occupancy in leguminous plants have shown that β-rhizobia outcompete α-rhizobia for infection in different *Mimosa* species (Elliott et al., 2009; Melkonian et al., 2014). In addition, a recent study from our group identified *Paraburkholderia phymatum* LMG 21445^T^ (=STM 815^T^) as a highly competitive symbiont that, when in competition with other β-rhizobia, was predominant in nodules of three out of four different legumes (Lardi et al., 2017). In that study, *P. phymatum* was also shown to produce higher amounts of exopolysaccharides (EPSs) and was the most competitive strain on plates, suggesting that inhibition of competitor growth was an important factor behind the successful competitiveness of *P. phymatum* (Lardi et al., 2017). Direct inhibition of bacteria growth can be achieved *via* secretion systems, which rely on structural components in the membrane to secrete effector proteins into the competitor cell (Stubbendieck and Straight, 2016; Bernal et al., 2017b). There are different types of such secretion systems in bacteria, including the newly described contact dependent type VI secretion systems (T6SSs), which are exclusively found in Gram-negative bacteria. T6SS can target either prokaryotic or eukaryotic cells (Schwarz et al., 2010; Angus et al., 2014; Ho et al., 2014). The structure and assembly of the T6SS complex has been determined for *Vibrio cholerae, Pseudomonas aeruginosa*, and *Escherichia coli* species (Basler et al., 2012, 2013; Shneider et al., 2013; Ho et al., 2014; Basler, 2015; Kudryashev et al., 2015; Vettiger and Basler, 2016; Vettiger et al., 2017). These studies showed that some structural elements of the T6SS share similarities with a phage tail spike (Records, 2011; Silverman et al., 2012; Ho et al., 2014) and also with the type IV secretion system T4SSb (Ma et al., 2009; Durand et al., 2012). A cluster of genes (*tss – t*ype *s*ix *s*ecretion) encodes the structural components of T6SS and functions as follow: the baseplate (TssA, E, F, G, K) and the membrane complex (TssJ, L, M), both anchored to the inner membrane, recruit the spike VgrG (*tssI* gene), which is attached to effectors; in the cytosol the sheath (TssB, C) polymerizes around the tube (Hcp, *tssD* gene); a contraction movement of the sheath expels the tube (Hcp) outside the cell, delivering the VgrG carrying effectors into the target cell. After the attack, ClpV (*tssM* gene), which is also attached to the membrane, uses ATP to reassemble the structure (Silverman et al., 2012; Ho et al., 2014; Alcoforado Diniz et al., 2015; Brunet et al., 2015; Gallique et al., 2017a). Phylogenetic reconstruction, using different genes of the T6SS cluster, revealed that most of the T6SSs are acquired by lateral gene transfer, and that small differences in regulation can lead to specificity and adaptation to different environments (Boyer et al., 2009).

Several plant-associated bacteria harbor genes coding for the T6SS in their genomes. The first description of a functional T6SS was in the symbiont *Rhizobium leguminosarum* (Bladergroen et al., 2003) where the system was shown to be important for the interaction with the plant host (Bladergroen et al., 2003; Nelson and Sadowsky, 2015; Ryu, 2015). On the other hand, in non-symbiotic plant-associated bacteria, T6SS was shown to be mainly involved in inter- or intra-bacterial competition. For instance, the biocontrol strain *Pseudomonas putida* KT2440 uses a T6SS for competition in soil and for protection of *Nicotiana benthamiana* plants from *Xanthomonas campestris* infection (Bernal et al., 2017a). In the plant pathogen *Pantoea ananatis* a T6SS mutant showed less virulence on onion plants and was affected in interbacterial competition against several bacteria (Shyntum et al., 2015). In *Agrobacterium tumefaciens*, competition against *Pseudomonas aeruginosa* inside a plant host (*N. benthamiana*) presented a T6SS-dependent advantage to *A. tumefaciens* (Ma et al., 2014).

In this work, we elucidated the role of two T6SS systems in the nitrogen-fixing and symbiotic strain *P. phymatum*. *P. phymatum* STM 815^T^ was isolated by Moulin et al. (2001) and was described by Vandamme et al. (2002) under the accession number LMG 21445^T^. The genome of *P. phymatum* STM 815^T^ has been sequenced (Moulin et al., 2014) and is available in NCBI under the following accession numbers for the replicons (NC_010622.1 for chromosome 1, NC_010623.1 for chromosome 2, NC_010625.1 for plasmid pBPHY01, and NC_010627.1 for the symbiotic plasmid pBPHY02). We show here that *P. phymatum* utilizes both T6SS systems to outcompete other β-rhizobial strains *in vitro* and to compete for legume root infection.

## Results

### Identification of Two Complete T6SS Clusters on the *P. phymatum* Megaplasmid pBPHY01

**FIGURE 1:**
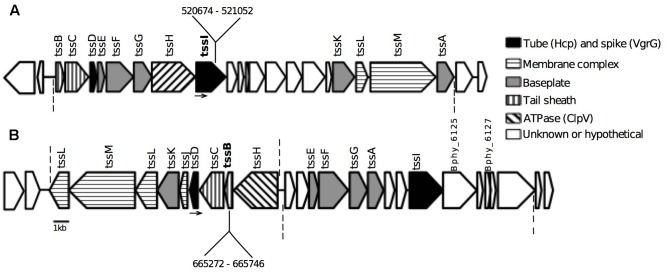
Type VI secretion system (T6SS) clusters on megaplasmid pBPHY01 of *Paraburkholderia phymatum* LMG 21445^T^. Nomenclature of the ORFs is according to the Burkholderia Genome Database (burkholderia.com). **(A)** T6SS-b cluster (position 510771–535444), structural elements coded between ORFs Bphy_5978 (BPHY_RS29885 – *tssB*) and Bphy_5997 (BPHY_RS29980 – *tssA*). **(B)** T6SS-3 cluster (position 654398–678783), structural elements coded between ORFs Bphy_6107 (BPHY_RS30485 – *tssL*) and Bphy_6124 (BPHY_RS30565 – *tssI*). The putative structural functions are marked in colors. Dashed lines indicate the beginning and the end of the putative operons based on preliminary RNA-seq data (Lardi et al., unpublished). Bphy_6125: (BPHY_RS38880) peptidase M23. Bphy_6127: PAAR repeat-containing protein. In bold are shown the genes mutated by insertion and the numbers indicate the position of the insertions (see “Materials and Methods”; Supplementary Table S1 and **Table 1**). Arrows indicate the external primers used for confirmation of the insertion (Bphy_5985For for the mutation in *tssI*-b gene and Bphy_6112Rev for the mutation in the *tssB*-3 gene – Supplementary Table S1).

A BLAST search on the *P. phymatum* megaplasmid pBPHY01 sequence (GenBank sequence: CP001045.1) (Moulin et al., 2014) using the T6SS1 and T6SS2 of *B. thailandensis* (Schwarz et al., 2010) identified two complete T6SS clusters within pBPHY01. **Table 1** lists the T6SS structural elements of both clusters and compares the amino acid sequences of the components. The low similarity between the components of both clusters suggests that the T6SS systems work independently of each other. The gene coding for the TssJ protein, which is part of the membrane complex, is present only in one of both clusters (**Figure 1**). Besides the cluster-encoded components, other T6SS related genes are found elsewhere in the *P. phymatum* genome, including six extra copies of the spike *tssI*-VgrG (Bphy_0023/BPHY_RS00115, Bphy_1932/BPHY_RS09825, Bphy_3640/BPHY_RS18400, Bphy_5197/BPHY_RS26040, Bphy_5744/BPHY_RS28740, and Bphy_7022/BPHY_RS34870). Both clusters contain, beside all structural *t*ype *s*ix *s*ecretion genes (*tssA-M*), several ORFs with unknown functions (**Figure 1**). According to a previous study by Angus et al. (2014), which presented a phylogenetic analysis based on proteins encoded by *tss* clusters of several *Burkholderia* strains, *P. phymatum* first *tss* cluster is a T6SS-b cluster (**Figure 1A**) and the second cluster a T6SS-3 (**Figure 1B**). An ORF (Bphy_5974/BPHY_RS29865), which is located upstream of the T6SS-b-type cluster, encodes a serine/threonine kinase that is involved in post-translational regulation of T6SS expression in *P. aeruginosa* (Casabona et al., 2013). The gene downstream of Bphy_5985 (BPHY_RS29920 *– tssI*), which codes for a VgrG effector, encodes a hypothetical protein (Bphy_5986/BPHY_RS29925) with an antitoxin domain (COG 2849) that may be required for immunity against self-killing. An ORF (Bphy_6117/BPHY_RS30535) coding for an accessory component, TagJ, is located within the T6SS-3 cluster. In *P. aeruginosa* TagJ, together with the product of *tssH* (ClpV), is involved in the recycling of the sheath (TssB, C) (Boyer et al., 2009; Lossi et al., 2012). The genomic region surrounding the *P. phymatum* T6SS-3 cluster contains several elements previously shown in other bacteria to be involved in the functioning of the system, such as a PAAR repeat-containing protein (Bphy_6127) (Shneider et al., 2013; Cianfanelli et al., 2016) and a putative peptidase M23 (Bphy_6125/BPHY_RS38880) (Shyntum et al., 2015; Weber et al., 2016). From the genomic organization and from our preliminary RNA-seq data on *P. phymatum* grown in free-living conditions, we suggest that both clusters are organized in operons (**Figure 1**) (Lardi et al., unpublished data).

**Table 1:** Comparison between amino acid sequences of the components of both *Paraburkholderia phymatum* LMG 21445^T^ type VI secretion systems (T6SSs) (T6SS-b and T6SS-3) (see **Figure 1**).

T6SS component	Function	Cluster T6SS-b	Cluster T6SS-3	% Identity (% similarity)
TssA	Baseplate	Bphy_5997 (BPHY_RS29980)	Bphy_6121 (BPHY_RS30555)	22 (36)
TssB	Tail sheath	Bphy_5978 (BPHY_RS29885)	**Bphy_6114** (BPHY_RS30520)	49 (68)
TssC	Tail sheath	Bphy_5979 (BPHY_RS29890)	Bphy_6113 (BPHY_RS30515)	48 (68)
TssD (Hcp)	Hcp Tube	Bphy_5980 (BPHY_RS29895)	Bphy_6112 (BPHY_RS30510)	25 (46)
TssE	Baseplate	Bphy_5981 (BPHY_RS29900)	Bphy_6118 (BPHY_RS30540)	32 (51)
TssF	Baseplate	Bphy_5982 (BPHY_RS29905)	Bphy_6119 (BPHY_RS30545)	31 (47)
TssG	Baseplate	Bphy_5983 (BPHY_RS29910)	Bphy_6120 (BPHY_RS30550)	28 (41)
TssH	ATPase	Bphy_5984 (BPHY_RS29915)	Bphy_6115 (BPHY_RS30525)	46 (60)
TssI (VgrG)	VgrG Spike	**Bphy_5985** (BPHY_RS29920)	Bphy_6124 (BPHY_RS30565)	33 (48)
TssJ	Membrane complex	Not present	Bphy_6111 (BPHY_RS30505)	Not accessed
TssK	Membrane complex	Bphy_5994 (BPHY_RS29965)	Bphy_6110 (BPHY_RS30500)	29 (47)
TssL	Membrane complex	Bphy_5995 (BPHY_RS29970)	Bphy_6109/6107 (BPHY_RS30495/BPHY_RS30485)	25 (49)/24 (39)
TssM	Membrane complex	Bphy_5996 (BPHY_RS29975)	Bphy_6108 (BPHY_RS30490)	21 (37)

*The genes mutated in this study (in bold) are indicated in **Figure 1** (*tssI* from T6SS-b, and *tssB* from T6SS-3). The identification of the components is given with two Locus Tags (old and new NCBI Locus Tag in brackets) taken from the Burkholderia Genome Database (burkholderia.com)*.

According to Angus et al. (2014), cluster-types T6SS-b and T6SS-3 are not associated with the pathogenicity of *Burkholderia* species.

### Phenotypic Analysis of the *P. phymatum* T6SS Mutant Strains

**FIGURE 2:**
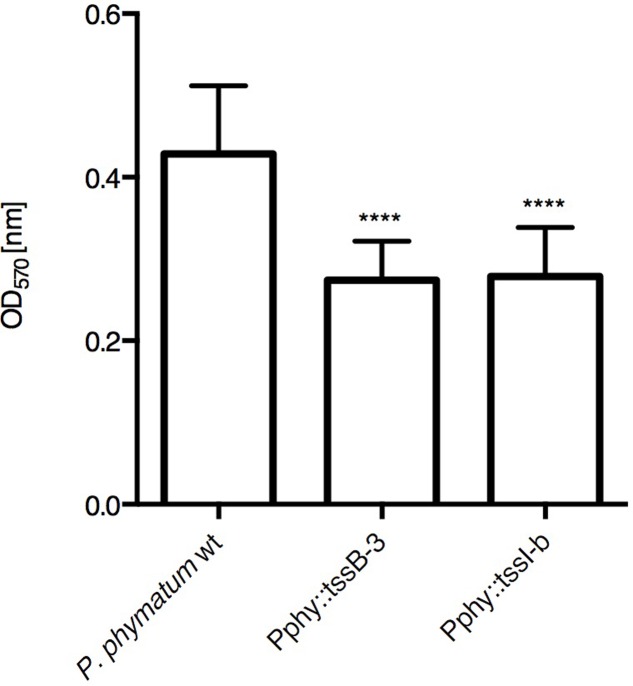
Biofilm formation in the *P. phymatum* strains. Biofilm formation was performed in 96-well plates. Each strain was measured in biological triplicates and eight wells per replica. ANOVA, Tukey’s test. ^∗∗∗∗^*p* ≤ 0.0001.

The gene *tssI* (Bphy_5985/BPHY_RS29920 position 519599–521524) from the cluster T6SS-b and *tssB* (Bphy_6114/BPHY_RS30520 position 665221–665754) from the cluster T6SS-3 were used as targets for the construction of insertional mutants, yielding the strains Pphy::tssI-b and Pphy::tssB-3, respectively. These genes were chosen because of their role as T6SS structural components and for being essential for the proper function of the secretion system (Boyer et al., 2009; Cianfanelli et al., 2016). The growth of the two *P. phymatum* T6SS mutant strains in rich media and minimal media (see “Materials and Methods”; Supplementary Figure S1) showed no difference compared to the wild-type, suggesting that the mutations do not affect the development of the bacteria. Phenotypes important for interaction with the plant host, such as EPS production and biofilm formation were tested in the T6SS mutant strains. Both phenotypes were previously shown to be dependent on a functional T6SS in *Pseudomonas fluorescens* (Decoin et al., 2015; Gallique et al., 2017b) in some *Burkholderia* species (Aubert et al., 2008) and in *Ralstonia solanacearum* (Zhang et al., 2014). In contrast, in *P. phymatum* EPS production was not influenced by the T6SSs (Supplementary Figure S2) as both mutant strains were able to produce as much EPS on YEM plates containing 0.06% yeast extract as the wild-type strain. On the other hand, biofilm formation was significantly decreased in the mutant strains, when compared to the wild-type (**Figure 2**).

In order to verify differences in the symbiotic performance of mutant strains compared to the wild-type, the strains were inoculated on the legume cowpea (*Vigna unguiculata*). After 3 weeks, the nodulated roots were used to determine the number and dry weight of the nodules as well as the nitrogenase activity. The overall parameters analyzed did not show any significant differences between the mutant strains and the wild-type (**Table 2**). Although the mutant strain Pphy::tssB-3 presented higher values than the *P. phymatum* wild-type strain in all the parameters tested (nodule number, nodule dry weight and nitrogenase activity), these differences were not statistically significant.

**Table 2:** Symbiotic properties of the T6SS mutant strains.

Parameter	Bacterial strains		
	*P. phymatum* LMG21445^T^	Pphy::tssI-b	Pphy::tssB-3
Nitrogenase [% (C_2_H_4_ C_2_H_2_^-1^) g^-1^ min^-1^]	0.46 ± 0.08	0.42 ± 0.12	0.55 ± 0.21
Dry weight nodules [mg]	0.17 ± 0.07	0.12 ± 0.06	0.19 ± 0.1
Nodule number	7.67 ± 3.79	8.75 ± 3.28	10.75 ± 3.92

*Values ± SD (standard deviation, ANOVA, Tukey’s test with *p* ≤ 0.05). The average of the number and the dry weight of nodules in the inoculated cowpea plants is given as well as the specific nitrogenase activity calculated after the ARA (acetylene reduction assay), normalized by the dry weight of the nodules and acetylene incubation time*.

With the purpose of verifying whether the T6SS mutant strains are impaired in competition for nodulation, a mixture containing equal amounts of the *P. phymatum* wild-type and of each of the mutant strains was tested on cowpea. For this, bacteria were mixed 1:1 (10^5^ cells) and inoculated on germinated cowpea seeds. Nodules harvested after 3 weeks were surface sterilized and plated on selective media to quantify the proportion of mutant/wild-type in each plant. No significant difference in the amounts of nodules colonized by the wild-type or mutants were observed, suggesting that the mutations did not affect the competitiveness of *P. phymatum* (Supplementary Figure S3).

### Competition with β-Rhizobial Strains for Legume Infection

To verify if the T6SS mutants are affected in interbacterial competition, we selected the legume host cowpea, which we previously showed to be exclusively nodulated by the wild-type strain of *P. phymatum* (Lardi et al., 2017). In fact, 100% of nodules recovered from cowpea plants inoculated with a mixture of β-rhizobia, containing *P. diazotrophica*, *P. mimosarum*, *P. sabiae*, and *P. phymatum*, were identified as *P. phymatum.* We therefore tested the same mixture of β-rhizobia against the T6SS mutants Pphy::tssB-3 and Pphy::tssI-b. The seeds were inoculated with two different cell densities (100 or 10^5^ cells of each strain). The low density setting was the same as we used in our previous study (Lardi et al., 2017). The high density setting was thought to increase the contact between bacteria at the moment of infection.

**FIGURE 3:**
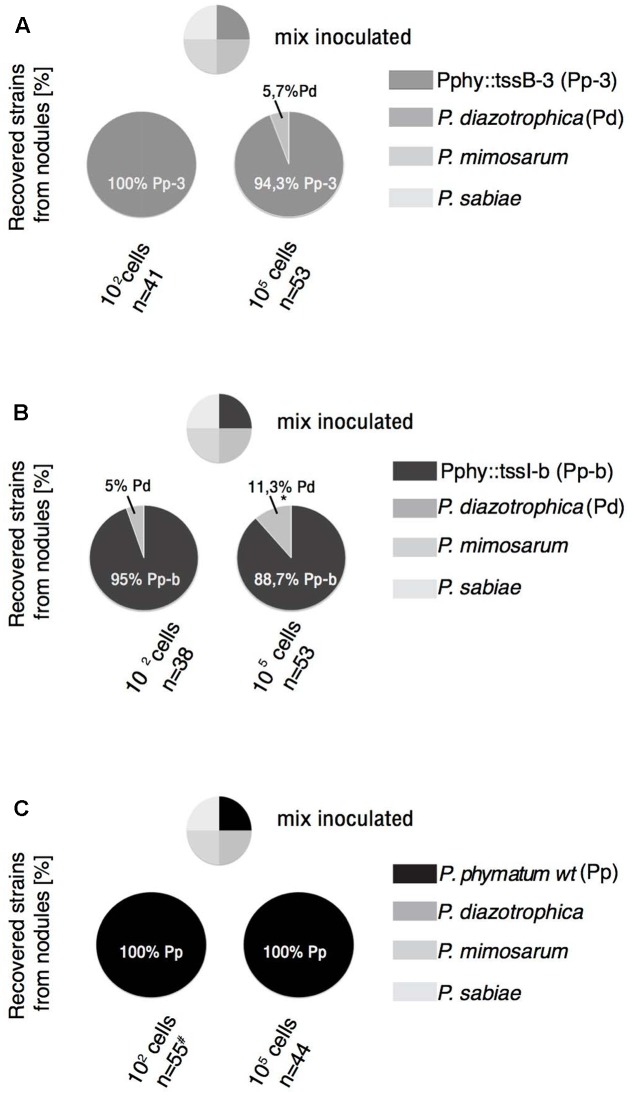
Effect of T6SS mutations in the competition of *P. phymatum* with other *Paraburkholderia* species *in planta*. The mixture containing 100 or 10^5^ cells of each of the four strains, *P. diazotrophica, P. mimosarum, P. sabiae* and Pphy::tssB-3 **(A)**, Pphy::tssI-b **(B)** or *P. phymatum* LMG 21445^T^ wild-type **(C)** was inoculated on seedlings of cowpea (*Vigna unguiculata*) and each nodule of the plants were harvested after 21 days and analyzed. Two independent replicates for each experiment and at least four plants were analyzed. The percentage of recovered strains is given. n, number of nodules. Two-tailed *z*-test comparing the proportion of *P. phymatum* obtained with the mixture containing each mutant against the one containing the wild-type. ^∗^*p* ≤ 0.05. # result from Lardi et al. (2017).

After 3 weeks of growth, all nodules from one plant were collected and subsequently analyzed for occupancy by plating on selective media (for the identification of *P. phymatum* mutant strains) and by using PCR with primers for the *recA* gene to identify the remaining strains (Mishra et al., 2012). While in the mixture containing 100 cells and the wild-type strain or the Pphy::tssB-3 mutant, 100% of the nodules were occupied by *P. phymatum* (**Figures 3A,C**), when the Pphy::tssI-b mutant was present in the mixture 5% of the nodules (2 out of 38) were occupied by *P. diazotrophica* (**Figure 3B**) and both nodules were located in the same plant root system. The plant assays with higher cell densities, where the *P. phymatum* wild-type was able to infect 100% of the nodules (**Figure 3C**), showed increased nodule colonization by strains other than the inoculated *P. phymatum* T6SS mutants. For the mixtures containing the Pphy::tssI-b or the Pphy::tssB-3 mutants, nodules infected by *P. diazotrophica* accounted for 11.3% (6 out of 53) and 5.7% (3 out of 53) of the total number of nodules analyzed, respectively (**Figure 3**). The nodules occupied by *P. diazotrophica* were found in the same root system of the nodules occupied by the T6SSs mutants, and in more than one plant. The other two strains of β-rhizobia tested (*P. mimosarum* and *P. sabiae*) were not identified in any of the experiments. These results suggest that T6SS is one of the factors responsible for the success of *P. phymatum* in infecting cowpea.

### Interbacterial *in Vitro* Competition Assays

**FIGURE 4:**
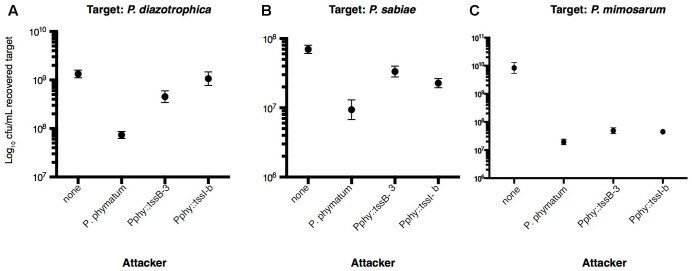
Interbacterial competition assay with *P. phymatum* (*dsRed* T6SS^+^) and T6SS mutants as attackers. The graphs show the recovered colony forming units (CFU) of the target bacteria: *P. diazotrophica gfp* **(A)**, *P. sabiae* **(B)**, and *P. mimosarum gfp* **(C)**. The dots represent the average of three independent experiments (+/– SEM). None: no attacker strain. Two-tailed *t*-test. **(A)** *p* = 0.0092 in the survival of targets against *P. phymatum* as attacker, *p* = 0.0445 against Pphy::tssB-3 and *p* = 0.6119 against Pphy::tssI-b. **(B)** *p* = 0.028 in the survival of the target against *P. phymatum*, *p* = 0.0282 against Pphy::tssB-3 and *p* = 0.0286 against Pphy::tssI-b. **(C)** *p* = 0.0139 in the survival of the target against *P. phymatum*, *p* = 0.1161 against Pphy::tssB-3 and *p* = 0.037 against Pphy::tssI-b.

To better understand the interaction between the bacterial strains tested in plant infection experiments, we set up interbacterial competition assays between *P. phymatum* (*dsRed*-T6SS^+^ and mutants) as attacker and the other β-rhizobia strains used in the plant experiments (*P. diazotrophica*-*gfp*, *P. mimosarum*-*gfp*, and *P. sabiae*) as targets strains (see “Materials and Methods” for details of strains used). The bacteria were mixed 1:1 (attacker:target) and co-cultured on a LB without salt plate for 24 h. The spot containing the competing bacteria was collected and the bacterial colony forming units (CFUs) were determined on selective plates. The survival of target bacteria is shown in **Figure 4**. When *P. phymatum* was tested in competition against *P. diazotrophica*, the *P. phymatum* T6SS system was revealed to play an important role. In competition against the *P. phymatum dsRed* T6SS^+^, 18.2-fold fewer cells of *P. diazotrophica* were recovered, compared to the strain grown alone (*p* ≤ 0.01) (**Figure 4A**). However, when the Pphy::tssI-b mutant was tested as attacker, *P. diazotrophica* survived as well as in the control without attacker (1.2-fold less from the CFU recovered compared to when *P. diazotrophica* was grown without attacker). The same tendency could be observed for competition of the Pphy::tssB-3 mutant against *P. diazotrophica* (2.9-fold decreased number of cells recovered compared to when *P. diazotrophica* was grown without attacker). Our results suggest that the *tssI* gene of T6SS-b and the *tssB* of the T6SS-3 are involved in the competition of *P. phymatum* against *P. diazotrophica* (**Figure 4A**). Likewise, the competition of *P. phymatum* against *P. sabiae* was also T6SS-dependent: in competition against the mutants, the survival of *P. sabiae* was significantly higher (Pphy::tssB-3 showing only 1.6-fold and Pphy::tssI-b 2.3-fold fewer recovered CFU compared to the *P. sabiae* strain grown without attacker) than what was observed for the assay including the *P. phymatum* wild-type, with 5.7-fold fewer cells of *P. sabiae* than when the strain grown alone (*p* ≤ 0.05) (**Figure 4B**). In contrast, the competition between *P. phymatum* and *P. mimosarum* showed that both the wild-type and the mutant strains significantly affected (*p* ≤ 0.05) the survival of *P. mimosarum* (171.4-fold, 186.7-fold, and 430.8-fold fewer cells against Pphy::tssB-3, Pphy::tssI-b and *P. phymatum dsRed* T6SS^+^, respectively, when compared to the CFU recovered by the strain alone). This result indicates that for competition between these two bacteria factors other than T6SSs are involved (**Figure 4C**). Taken together, these data show that both T6SSs of *P. phymatum* play an important role in the competition against other bacteria such as *P. diazotrophica* and *P. sabiae*.

## Discussion

In this work we describe the role of T6SSs in a symbiotic and nitrogen fixing β-rhizobia that interacts with a variety of leguminous plants. We have previously shown that *P. phymatum* LMG 21445^T^ outcompetes other β-rhizobia in plant infection of papilionoid legumes and *in vitro* on plates (Lardi et al., 2017). Here, we investigate the genetic mechanisms underlying the high level of competitiveness of *P. phymatum* by characterizing the importance of the T6SSs in this strain. T6SSs have been described in various Gram-negative bacteria and were shown to act against both prokaryotes and eukaryotes (Bingle et al., 2008; Filloux et al., 2008; Boyer et al., 2009; Basler et al., 2012, 2013; Alcoforado Diniz et al., 2015; Basler, 2015). Interestingly, the first report of a T6SS was in *Rhizobium leguminosarum*, where the wild-type strain was shown to be impaired in pea nodulation (Roest et al., 1997), while a mutation in the *imp* gene cluster (which was later identified as a T6SS) (Bladergroen et al., 2003) caused a nodulation phenotype. The authors could show that the impairment in pea nodulation was due to the secretion of proteins into the plant host cells (Bladergroen et al., 2003). Most of the research on T6SSs is done in pathogenic bacteria and the role of several effectors secreted through the system consists in the degradation of the cell wall of the attacked cell (Alcoforado Diniz et al., 2015). In pathogenic *Burkholderia*, T6SSs are used to attack both eukaryotic and prokaryotic target cells (Aubert et al., 2008; Schwarz et al., 2010, 2014; Burtnick et al., 2011; Angus et al., 2014). In the *P*. *phymatum* genome (Moulin et al., 2014) we identified two complete T6SS clusters. These T6SS clusters, according to the classification of Angus et al. (2014), are not associated with the pathogenicity of *Burkholderia* species (Schwarz et al., 2010, 2014; Burtnick et al., 2011). Moreover, the *P*. *phymatum* genome encodes eight VgrG proteins and only two of them are located inside a T6SS cluster.

Besides secreting effectors, T6SS are known to affect biofilm formation. For instance, the plant pathogen *Ralstonia solanacearum* possesses a T6SS gene cluster, which is not only important for pathogenicity in tomato plants but also has a strong effect on motility and biofilm formation (Zhang et al., 2014). In *Pseudomonas fluorescens* MFE01, a deletion mutant in the *tssC* gene, coding for the tail sheath component, was also impaired in biofilm formation (Gallique et al., 2017b). We found that both *P. phymatum* T6SS mutants showed a significantly decreased ability to form biofilms (**Figure 2**) while plant infection efficiency, at least for cowpea plants, was not affected (**Table 2**).

We previously showed that *P. phymatum* is highly competitive for nodulation of cowpea, where all nodules formed on the roots were infected by *P. phymatum*. In this study, we showed that using the same mixture of strains and the same growth conditions we described previously (Lardi et al., 2017) but with the *P. phymatum* T6SS insertional mutants instead of the wild-type, we have a different outcome in our nodulation competition assay (**Figure 3**). With the *P. phymatum* T6SS mutants in the bacterial mixture and a higher cell density inoculum (10^5^ cells), we were able to recover, besides *P. phymatum*, *P. diazotrophica* from the nodules. The latter is a β-rhizobial strain isolated from nodules of different *Mimosa* species from several geographical locations (Sheu et al., 2013) and its genome has recently been sequenced (Beukes et al., 2017). The results on the plant competition could be explained either by the impairment of the *P. phymatum* T6SS mutants to establish an efficient infection with the plant host (cowpea) or by a disadvantage to compete with other soil bacteria. Inspection of the recently sequenced *P. diazotrophica* and *P. mimosarum* genomes revealed the presence of a complete T6SS system also in these strains. The role of the T6SSs in these strains is currently unknown. Since the *P. phymatum* mutants constructed in our study were not impaired in the infection of cowpea (**Table 2**), we suggest that the *P. phymatum* T6SS plays a role in β-rhizobial competition for plant infection.

Even though the T6SS in bacterial pathogens was firstly described as usually important for the interaction with eukaryotic host cells, the role in interbacterial competition was shown to be the most common feature of T6SSs. Many reports, on different bacteria, have shown that the system is successfully used to kill bacteria. Plant-associated bacteria belonging to the genera *Agrobacterium*, *Pantoea*, and *Pseudomonas* (Ma et al., 2009, 2014; Shyntum et al., 2015; Bernal et al., 2017a) were shown to utilize T6SSs to outcompete other members of the soil community (Bernal et al., 2017a). Here we show that the T6SS clusters present in *P. phymatum* are important for interbacterial competition of the strain *in vitro* and *in vivo*. In contrast to what has been shown previously with *R. leguminosarum* (Bladergroen et al., 2003; Ryu, 2015), *P. phymatum* T6SSs do not affect the establishment of a symbiosis with the plant host. In term of nodule dry weight as well as nodule number and nitrogenase activity, cowpea plants infected with the T6SS mutant strains behaved similar to the ones inoculated with the wild-type. Preliminary tests suggested similar results using common bean as host legume (data not shown). We show here that *P. phymatum* uses T6SSs to outcompete *P. diazotrophica* and *P. sabiae*. The survival rate of these two strains (shown in **Figure 4** as the recovered CFU) when attacked by a *P. phymatum* T6SS mutant was significantly higher relative to survival against the wild-type and comparable with the survival rate without attacker. We cannot rule out that the slight differences in the phenotype observed with both mutants are due to the gene disrupted (*tssB* in cluster T6SS-3 and *tssI-b* in cluster T6SS-b) or to a polar effect caused by the insertion of the plasmid. Taken together, the results of the *in vitro* assays are in good agreement with the competition experiments for legume nodulation. The reason why *P. diazotrophica* is outcompeting the other β-rhizobia when the *P. phymatum* wild-type was replaced with the T6SS mutants is unclear.

Interestingly, killing of *P. mimosarum* by *P. phymatum* seemed independent of the T6SSs (**Figure 4C**). Maybe other *P. phymatum* competition traits, such as the contact-dependent inhibition system (CDI) or antibiotic production (Aoki et al., 2010; Stubbendieck and Straight, 2016) could be responsible for the competitive advantage of *P. phymatum* over *P. mimosarum*. It is noteworthy that *P. mimosarum* was shown to be more effective for N_2_-fixation and nodulation than *P. phymatum* on the cognate β-rhizobial legume host *Mimosa pudica* (Lardi et al., 2017). Therefore, T6SS-mediated competition prior to infection may be less important for β-rhizobial competition in the case of *M. pudica* infection.

This study reports on the initial characterization of the role of the T6SSs in the β-rhizobial strain *P. phymatum*. We demonstrate that in this bacterium the T6SS is involved in biofilm formation and plays an important role in competition against other β-rhizobial strains *in vitro* and *in vivo* on the papilionoid legume cowpea as host. Our results support a scenario where T6SS-mediated competition is needed for selective killing of competitors, which in turn will have a strong impact on which of the present soil bacteria will infect the roots of a given plant host. Further work will be required to elucidate the contribution of each components of the systems and to figure out how *P. phymatum* T6SSs are activated in a natural environment.

## Materials and Methods

### Bacterial Strains and Growth Conditions

*Paraburkholderia* strains (described in the Supplementary Table S1) were cultivated under aerobic conditions at 30°C in LB medium without salt (10 g/L tryptone and 5 g/L yeast extract), and supplemented with appropriate antibiotics. *E. coli* strains (Supplementary Table S1) for cloning and helper strain for tri-parental mating were multiplied in LB (10 g/L tryptone, 5 g/L yeast extract, and 4 g/L NaCl). For plant nodulation tests, for tri-parental mating and biofilm formation the cultures were washed in defined buffered AB-minimal medium (Clark and Maaloe, 1967) without nitrogen and with 10 mM sodium citrate as the carbon source while for EPS and interbacterial competition assays the cells were washed in LB medium without salt.

### Construction of Mutant Strains

A fragment of 378 bp from the gene Bphy_5985 (BPHY_RS29920) (*tssI*-b, 519599–521524) and of 456 bp from the gene Bphy_6114 (BPHY_RS30520) (*tssB*-3, 665221665754) was amplified from the genomic DNA of *P. phymatum* with primers that introduced *Eco*RI sites on the extremities (tssI-bFor and tssI-bRev; tssB-3For and tssB-3Rev – Supplementary Table S1). The PCR products were purified with the Qiagen^®^ PCR purification kit, and digested with *Eco*RI (NEB). The suicide vector pSHAFT2 (Shastri et al., 2017) was also digested with *Eco*RI, and used for a ligation with the digested PCR fragments using T4 DNA Ligase from Roche^®^. The ligation reaction was transformed in chemically competent *E. coli* CC118pir cells, and the insertion of the fragments was confirmed by sequencing with the primer pSHAFTseqFor (Supplementary Table S1). The constructs were introduced in *P. phymatum* by tri-parental mating using *E. coli* DH5α pRK2013 as helper. The transconjugants were purified twice in ABC minimal media with chloramphenicol 80 μg/ml. The correct insertion was confirmed by amplification with external primers annealing in the genome of *P. phymatum* (Bphy_5985For position 519929 in the case of *tssI*-b and Bphy_6112Rev position 663149 in the case of *tssB*-3, **Figure 1**) with the pSHAFTseqFor primer (Supplementary Table S1). The resulting strains are called Pphy::tssI-b and Pphy::tssB-3.

For the generation of the tagged *P. diazotrophica, P. mimosarum*, and *P. phymatum* strains, tri-parental mating was performed, using *E. coli* DH5α containing pRK2013 as helper, and *E. coli* with the plasmids pBAH8 (Huber et al., 2002) and pIN62 (Vergunst et al., 2010) as donors. The resulting strains were used in the *in vitro* competition assays.

### Phenotypic Characterization of the T6SS Mutant Strains

In order to verify the phenotype of the *P. phymatum* mutants, different aspects of the physiology were determined and compared to the wild-type strain. The growth of the strains was analyzed in rich media (LB without salt) and minimal media (ABC). For the assessment of EPS production, a cotton swab was plunged in a bacterial suspension with an OD_600_ 0.5 and streaked on modified YEM plates (1% mannitol, 0.06% yeast extract) (Richau et al., 2000). The plates were kept for 72 h at 30°C. Biofilm formation was detected in cell culture plate, 96-well, polystyrene (Sarstedt), inoculated with AB-minimal medium (Clark and Maaloe, 1967) and 10 mM of sodium citrate as carbon source, as described previously (Huber et al., 2002; Pessi et al., 2013; Lardi et al., 2015) with a longer incubation time (72 h).

### Plant Growth Conditions and Nitrogenase Activity

Cowpea (*Vigna unguiculata* (L.) Walp. cv. Red Caloona, kindly provided by Prof. Hans-Martin Fischer –ETH Zurich) surface sterilized seeds (Klonowska et al., 2012) were placed on 0.8% agar plates and incubated in the dark at 30°C for 24–36 h. Germinated seedlings were introduced into autoclaved yoghurt-jar containing vermiculite (VTT-Group, Muttenz, Switzerland) and 170 ml diluted Jensen medium (Hahn and Hennecke, 1984). Direct seed inoculation with the desired bacterial strains (10^5^ cells) or with the bacterial mixtures (10^2^ and 10^5^ cells) was done. The plants were grown as follows in a growth chamber: 22°C (night) and 27°C (day); 12 h light (200 μMol intensity); humidity 60%. The plants were harvested 21 days post-infection (dpi), and the determination of symbiotic properties (nodule number, nodule dry weight and determination of nitrogenase activity) was done according to Göttfert et al. (1990). ARA (acetylene reduction assay) was done to determine the specific nitrogenase activity of each plant. For that, 1 ml of acetylene (PanGas, Zürich, Switzerland) was injected in 50 ml tubes (Infochroma AG, Zug, Switzerland) containing the root of the plant to be analyzed. Gas chromatography (Agilent Technologies, 6850 Network GC System) (Koch et al., 2010) was performed by injecting 25 μl of the incubated gas. The specific nitrogenase activity was obtained by taking the relative area of ethylene produced, normalized by nodule number, dry weight and incubation time. At least three plants per experiment were tested for single infection. Two independent experiment were performed.

### Competition Assays *in Planta*

The strains for inoculation were grown until stationary phase in LB medium without salt. After washing the cultures twice in minimal medium (AB) without nitrogen, the OD_600_ of each culture was adjusted to 0.01. The CFU was verified by plating on LB without salt plates. For the first test, the cultures were diluted to 10^2^ cells, and for the second test to 10^5^ cells. The bacterial mixture containing *P. phymatum* (wild-type, Pphy::tssI-b or Pphy::tssB-3), *P. diazotrophica, P. mimosarum, P. sabiae* in equal amounts was immediately applied on cowpea germinated seeds (1 ml/seedling). The mixture containing the *P. phymatum* wild-type was tested only with an inoculum of 10^5^ cells. Two independent experiments were performed per test and per experiment a minimum of four plants of cowpea were analyzed. After 3 weeks of incubation, the roots were surface sterilized by plunging for 10 s in 100% ethanol, then 3 min in 2.5% sodium hypochloride and finally washed five times with sterile deionized water (Vincent, 1970). All the nodules present on the roots of the plants were collected individually and placed on an Eppendorf tube containing 100 μl LB without salt with 50% glycerol. The nodules were then individually crushed and plated onto two solid LB medium without salt, one of them containing chloramphenicol for the selection of the mutant *P. phymatum* strains. Single colonies grown on the plate without antibiotic were tested via colony PCR with degenerated *recA* primers (recABurk1_F and recABurk1_R; Supplementary Table S1), which generate a product with all the four *Paraburkholderia* strains used in this study (Mishra et al., 2012). The obtained PCR product was purified and sequenced (at Microsynth AG, Switzerland).

### Competition Assays *in Vitro*

Interbacterial competition was tested by killing assays on plate, as described by Shyntum et al. (2015), with the following modifications. *P. phymatum* strains (wild type-*dsRed* tagged T6SS^+^ and T6SS mutants) were used as attackers, while *P. diazotrophica* (*gfp* tagged), *P. mimosarum* (*gfp* tagged), and *P. sabiae* were target bacteria. Bacteria were grown until stationary phase in LB medium without salt, washed once with the same media and normalized to OD_600_ 0.1 (Supplementary Figure S4). The inoculum corresponded to a CFU of 10^8^ for each strain. The strains were mixed 1:1 (attacker:target) and 5 μl were spotted on LB plates without salt and incubated 24 h at 30°C. After incubation, the bacteria were recovered from the plate, resuspended in 1 ml of the same media and serial dilution (10^-3^ until 10^-8^) was plated. CFU were counted on LB without salt plates and on selective plates, i.e., LB medium without salt containing chloramphenicol (for *P. phymatum dsRed* and for the *P. phymatum* mutant strains), and containing gentamycin (for *P. mimosarum* and *P. diazotrophica gfp* tagged strains). *P. sabiae* recovery was counted by subtracting the number of attacker colonies grown in chloramphenicol with the ones grown without antibiotic.

### Statistical Analyses

Prism software version 6.0c was used for all the statistical analyses. Biofilm formation and plant test phenotypes (nodule number, nodule dry weight and specific nitrogenase activity) were analyzed using one-way ANOVA with *p* ≤ 0.05. Interbacterial competition experiments were analyzed with two-tailed *t*-test with *p* ≤ 0.05, while the proportions of bacteria after competition on cowpea plants were analyzed by *z*-test (chi-square) with *p* ≤ 0.05.

## Author Contributions

SdC: Conceived and designed the experiments, performed the experiments, analyzed the data, wrote the paper. ML: Performed the experiments, Analyzed the data. AG: Performed the experiments. LE: Analyzed the data. GP: Conceived and designed the experiments, analyzed the data, wrote the paper.

## Conflict of Interest Statement

The authors declare that the research was conducted in the absence of any commercial or financial relationships that could be construed as a potential conflict of interest.
