# Supplementary material for: Mutations in Two Paraburkholderia phymatum Type VI Secretion Systems Cause Reduced Fitness in Interbacterial Competition
Source: Front Microbiol. 2017 Dec 12;8:2473. doi: 10.3389/fmicb.2017.02473 (PMC5732942; doi:10.3389/fmicb.2017.02473)
Supplement: Supplementary file 1 [file Table_1.PDF]

Supplementary Table S1: Bacterial strains, plasmids and oligonucleotides used in this study.

		**References**
Paraburkholderia		
P. diazotrophica LMG26031^T	Isolated from *Mimosa* spp.	(Sheu et al., 2013)
P. mimosarum LMG23256^T	Isolated from *Mimosa* spp.	(Chen et al., 2006)
P. phymatum LMG21445^T	Isolated from *Mimosa* spp.	(Moulin et al., 2001, Vandamme et al., 2002)
P. phymatum dsRed	*P. phymatum* LMG21445^T carrying the pIN62, Cm^R	This study
P. sabiae LMG24235^T	Isolated from *Mimosa caesalpiniifolia*	(Chen et al., 2008)
Pphy::tssI-b	*P. phymatum* with pSHAFT cassette disrupting the gene *tssI*-b (Bphy_5985), Cm^R	This study
Pphy::tssB-3	*P. phymatum* with pSHAFT cassette disrupting the gene *tssB*-3 (Bphy_6114), Cm^R	This study
P. mimosarum gfp	*P. mimosarum* carrying pBAH8, Gm^R	This study
P. diazotrophica gfp	*P. diazotrophica* carrying pBAH8, Gm^R	This study
Escherichia coli		
cc118 λ–pir	Δ(ara-leu) *araD* Δ*lacX*74 *galE galK phoA*20 *thi1 rpsE rpoB argE*(Am*) recAl* λ *pir*; Strep^R	(Herrero et al., 1990)
Top10	Δ*lacX74 ara*Δ139Δ(ara-leu)	Invitrogen ®
**Plasmids**		
pRK2013	Helper plasmid, Km^R	(Figurski and Helinski, 1979)
pSHAFT 2	Broad-host-range suicide plasmid, mobilisable for conjugation; Cm^R	(Shastri et al., 2017)
pSHAFTtssI-b	pSHAFT 2 carrying a 378bp fragment of the *tssI*-b gene on the *Eco*RI site	This study
pSHAFTtssB-3	pSHAFT 2 carrying a 456bp fragment of the *tssB*-3 gene on the *Eco*RI site	This study
pBAH8	pBBR1MCS-5 containing $P_{A1/04/03}$-*gfp mut3*-T_o-T_1	(Huber et al., 2002)
pIN62	*ori*$_{pBBR}$ *mob*$^+$, Cm^r, *dsRed*	(Vergunst et al., 2010)
**Oligonucleotides**		
tssI-bFor (position 520667)	TTTGAATTCGGTACGCATACGGCAAAAGT	This study
tssI-bRev (position 521044)	TTTGAATTCGGAATTGAGCTTGTCCTCCA	This study
Bphy_5985For (position 519929)	CGTGTCATCTGGCAGAACCT	This study
tssB-3For (position 665746)	TTTGAATTCCAGCAGTTCGCAGAAGTTCA	This study
tssB-3Rev (position 665291)	TTTGAATTCAGAAGCGAGGGATCCTGAAG	This study
Bphy_6112Rev (position 663149)	TTGTTGCCCTTGATGTCGTA	This study
pSHAFTseqFor	CTTCAGCTGATGTGTGATAACATACT	Agnoli, K. unpublished
recABurkF	GATCGARAAGCAGTTCGGCAA	(Mishra et al., 2012)
recABurkR	TTGTCCTTGCCCTGRCCGAT	(Mishra et al., 2012)

Underlined sequences on the oligonucleotides represent restriction enzyme recognition sites. The genomic coordinates of the primers are given in brackets.

References:

Chen, W. M., De Faria, S. M., Chou, J. H., James, E. K., Elliott, G. N., Sprent, J. I., et al. (2008). *Burkholderia sabiae* sp. nov., isolated from root nodules of *Mimosa*

caesalpiniifolia. *Int. J. Syst. Evol. Microbiol.* 58**,** 2174-2179. doi: 10.1099/ijs.0.65816-0

Chen, W. M., James, E. K., Coenye, T., Chou, J. H., Barrios, E., De Faria, S. M., et al. (2006). *Burkholderia mimosarum* sp. nov., isolated from root nodules of *Mimosa* spp. from Taiwan and South America. *Int. J. Syst. Evol. Microbiol.* 56**,** 1847-1851. doi: 10.1099/ijs.0.64325-0

Figurski, D. H. & Helinski, D. R. (1979). Replication of an origin-containing derivative of plasmid RK2 dependent on a plasmid function provided in trans. *Proc. Natl. Acad. Sci. U S A* 76**,** 1648-1652.

Herrero, M., De Lorenzo, V. & Timmis, K. N. (1990). Transposon vectors containing non-antibiotic resistance selection markers for cloning and stable chromosomal insertion of foreign genes in gram-negative bacteria. *J. Bacteriol.* 172**,** 6557-6567.

Huber, B., Riedel, K., Kothe, M., Givskov, M., Molin, S. & Eberl, L. (2002). Genetic analysis of functions involved in the late stages of biofilm development in *Burkholderia cepacia* H111. *Mol. Microbiol.* 46**,** 411-426. doi: 10.1046/j.1365-2958.2002.03182.x

Mishra, R. P., Tisseyre, P., Melkonian, R., Chaintreuil, C., Miche, L., Klonowska, A., et al. (2012). Genetic diversity of *Mimosa pudica* rhizobial symbionts in soils of French Guiana: investigating the origin and diversity of *Burkholderia phymatum* and other beta-rhizobia. *FEMS Microbiol. Ecol.* 79**,** 487-503. doi: 10.1111/j.1574-6941.2011.01235.x

Moulin, L., Munive, A., Dreyfus, B. & Boivin-Masson, C. (2001). Nodulation of legumes by members of the beta-subclass of Proteobacteria. *Nature* 411**,** 948-950. doi: 10.1038/35082070

Shastri, S., Spiewak, H. L., Sofoluwe, A., Eidsvaag, V. A., Asghar, A. H., Pereira, T., et al. (2017). An efficient system for the generation of marked genetic mutants in members of the genus *Burkholderia*. *Plasmid* 89**,** 49-56. doi: 10.1016/j.plasmid.2016.11.002

Sheu, S. Y., Chou, J. H., Bontemps, C., Elliott, G. N., Gross, E., Dos Reis Junior, F. B., et al. (2013). *Burkholderia diazotrophica* sp. nov., isolated from root nodules of *Mimosa* spp. *Int. J. Syst. Evol. Microbiol.* 63**,** 435-441. doi: 10.1099/ijs.0.039859-0

Vergunst, A. C., Meijer, A. H., Renshaw, S. A. & O'callaghan, D. (2010). *Burkholderia cenocepacia* creates an intramacrophage replication niche in zebrafish embryos, followed by bacterial dissemination and establishment of systemic infection. *Infect. Immun.* 78**,** 1495-1508. doi: 10.1128/IAI.00743-09

Vandamme, P., Goris, J., Chen, W. M., De Vos, P. & Willems, A. (2002). *Burkholderia tuberum* sp. nov. and *Burkholderia phymatum* sp. nov., nodulate the roots of tropical legumes. *Syst. Appl. Microbiol.* 25**,** 507-512. doi: 10.1078/07232020260517634
