# Supplementary figures and images for: Mutations in Two Paraburkholderia phymatum Type VI Secretion Systems Cause Reduced Fitness in Interbacterial Competition
Source: Front Microbiol. 2017 Dec 12;8:2473. doi: 10.3389/fmicb.2017.02473 (PMC5732942; doi:10.3389/fmicb.2017.02473)

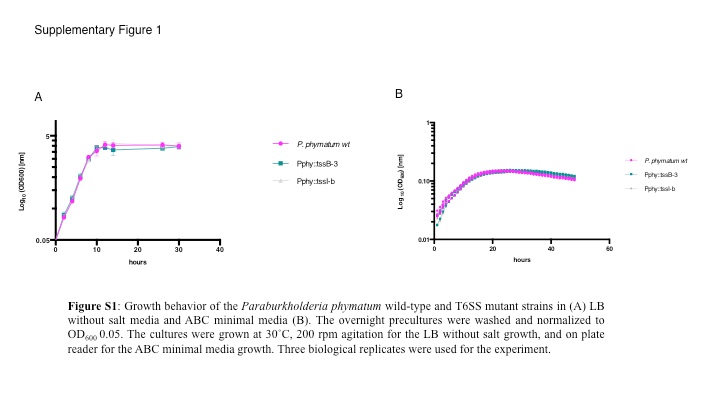

Supplement: Supplementary file 2 [file Image_1.PNG]

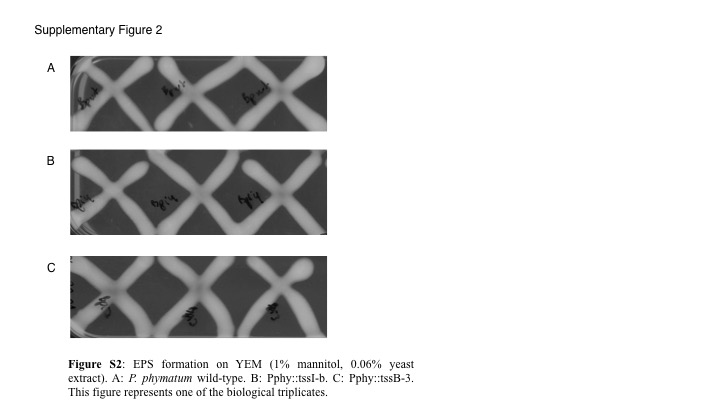

Supplement: Supplementary file 3 [file Image_2.JPEG]

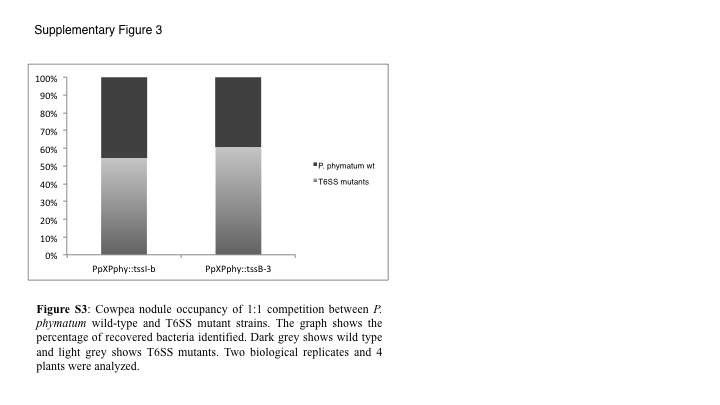

Supplement: Supplementary file 4 [file Image_3.JPEG]

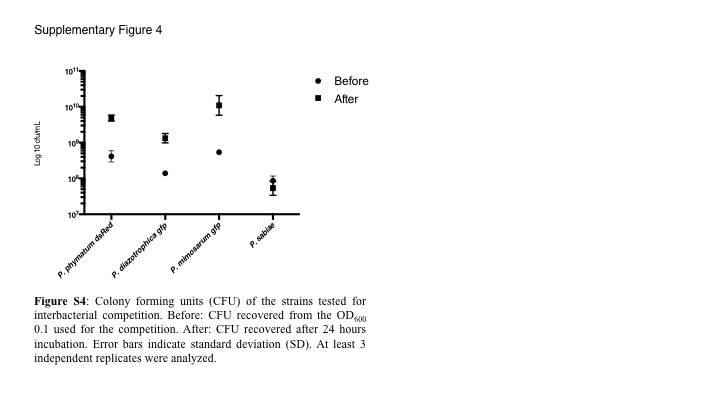

Supplement: Supplementary file 5 [file Image_4.jpg]
